# Supplementary material for: Tracking the Transcription Kinetic of SARS-CoV-2 in Human Cells by Reverse Transcription-Droplet Digital PCR
Source: Pathogens. 2021 Oct 2;10(10):1274. doi: 10.3390/pathogens10101274 (PMC8538813; doi:10.3390/pathogens10101274)
Supplement: Supplementary file 1 [file pathogens-10-01274-s001.zip › supple for conversion/pathogens-1383339-supplementary figures.pdf]

Supplementary Figure

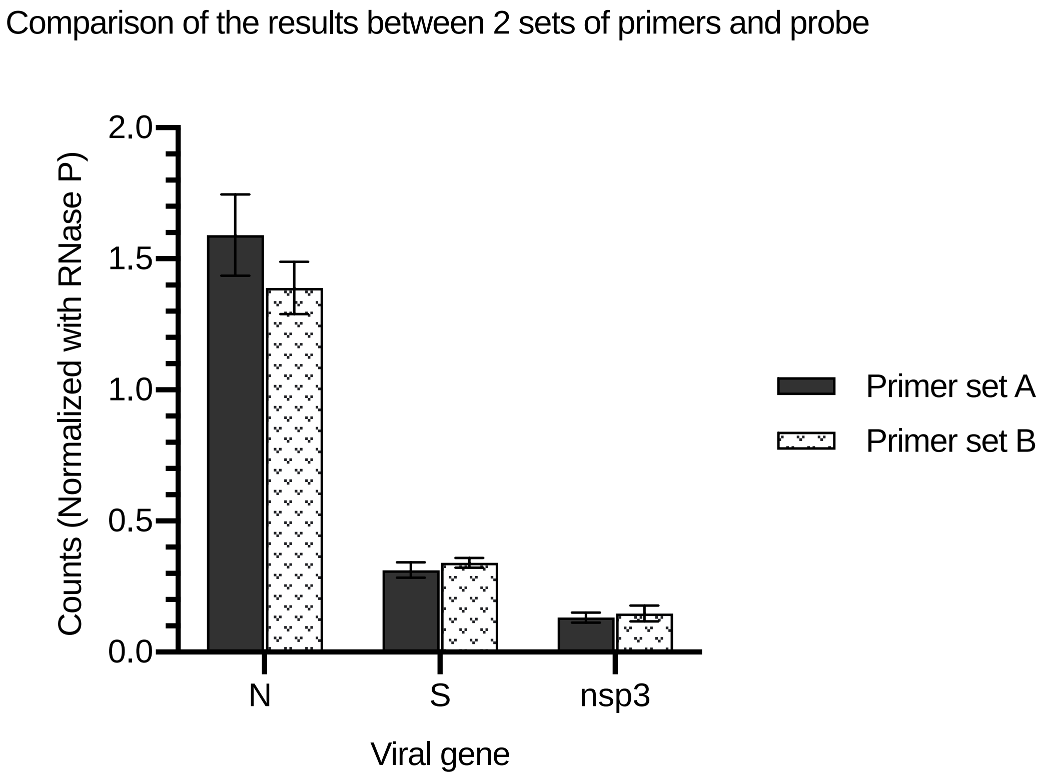

Figure S1. Primers and probes that designed on different conserve regions show no difference of results by ddPCR. Additional 3 sets of oligos and probes (labelled as set B) that target to different conserve regions of S, N and NSP3 were designed and used for ddPCR to compare the amplification efficiency. Human Caco-2 cells were infected by the SARS-CoV-2 at a moi of 0.01 and the total RNA was collected at 12 hours after infection. #:P>0.05.
